# Implementation by nurses of Hospital Admission Authorization in Birth Centers

**DOI:** 10.1590/0034-7167-2024-0404

**Published:** 2025-09-01

**Authors:** Antonio Rodrigues Ferreira, Raimundo Neto de Abreu e Brito, Adriano da Costa Belarmino, Fernanda Maria Carvalho Fontenele, Saiwori de Jesus Silva Bezerra dos Anjos, Luiza Jane Eyre de Souza Vieira

**Affiliations:** I Universidade Estadual do Ceará. Fortaleza, Ceará, Brazil; II Hospital e Maternidade Dr José Maria Fernandes Leitão. Novo Oriente, Ceará, Brazil; III Universidade de Fortaleza. Fortaleza, Ceará, Brazil

**Keywords:** Nurse Midwives, Birthing Centers, Professional Role, Professional Autonomy, Decision Making, Enfermeras Obstetrices, Centros de Asistencia al Embarazo y al Parto, Rol Profesional, Autonomía Professional, Toma de Decisiones

## Abstract

**Objectives:**

to report nurse-midwives’ autonomy in implementing Hospital Admission Authorization in Birth Centers.

**Methods:**

this is a case study in an intra-hospital Birth Centers in Ceará, Brazil, of events that occurred between 2018 and 2023. The organization of the description followed the stages of thematic analysis according to Nowell.

**Results:**

the autonomy and freedom of nurse-midwives’ action in care and management practices in labor, delivery and birth provide the professional category with a leading role in the obstetric scenario, highlighting its need to guarantee women’s rights and reduce maternal-fetal complications.

**Final Considerations:**

the issuance of Hospital Admission Authorization by nurse-midwives in Birth Centers represents a significant advance for nursing teams’ ethical and healthcare actions, contributing to effective results in maternal and child health and in achieving progress in advanced practice in the profession of nursing-midwifery.

## RESUMEN

**Objetivos:**

informar la autonomía de los enfermeros obstétricos en la implementación de la Autorización de Ingreso Hospitalario en Centros de Parto Natural.

**Métodos:**

se trata de un estudio de caso en un Centros de Parto Natural intrahospitalaria de Ceará, Brasil, de eventos ocurridos entre 2018 y 2023. La organización de la descripción siguió los pasos del análisis temático según Nowell.

**Resultados:**

la autonomía y libertad de acción del enfermero obstétrico en las prácticas de atención y gestión en el trabajo de parto, parto y parto otorgan el protagonismo de la categoría profesional en el escenario obstétrico y resaltan su necesidad de garantizar los derechos de las mujeres y reducir las complicaciones materno-fetales.

**Consideraciones Finales:**

la emisión de Autorización de Ingreso Hospitalario por parte del enfermero obstétrico del Centros de Parto Natural representa un avance significativo para el accionar ético y asistencial del equipo de enfermería, contribuyendo para resultados efectivos en la salud materno infantil y en el logro de avances en la práctica avanzada de la profesión de enfermería obstetra.

## INTRODUCTION

Ensuring maternal mortality rates of less than 70 per 100,000 live births remains one of the greatest challenges of the Sustainable Development Goals (SDGs) in the world, especially after the COVID-19 pandemic. In 2020, 800 women died every day worldwide, mainly due to preventable causes and unnecessary interventions during labor and birth, such as surgical delivery without indication. Approximately 95% of these deaths occurred in lowand middle-income countries, such as Brazil^(1)^.

From this perspective, healthcare models, such as the Stork Network Strategy and the Alyne Network, play a key role in guaranteeing rights, social justice, equity in access, care and assistance in maternal and child health in the country^(2,3)^. In the Childbirth and Birth component of the Stork Network and Alyne Network, Birth Centers (BCs) have been developing models of childbirth care that prioritize practices based on scientific evidence, environments and structures aimed at ensuring familiarity and comfort, a trained professional team and actions that guarantee women’s right to quality childbirth and birth, as well as their leading role and decision-making in care processes^(2)^.

In this context, nurse-midwives stand out as promoters of assistance, care and work management in BCs, encouraged by public health policies and by society. However, for this to occur, obstacles and obstacles related to professional and power disputes are still faced within the biomedical and hospital-centric model of care for childbirth and birth in force in the country^(4,5)^.

One of the essential skills for working in these spaces and overcoming obstacles is autonomy for health actions. Among them is the implementation of a Hospital Admission Authorization (HAA) for women in labor and delivery, which is legally permitted for nurse-midwives. HAA is a document that contains data about users of the Brazilian health system, which feeds the hospital and outpatient information systems to produce indicators^(4,6)^.

In this regard, the adopted concept of autonomy refers to skills and techniques, such as knowledge, absence of dependence on others, decision-making power, competency, self-governance, freedom, capacity for affection, judgment, self-control and responsibility^(7)^. The guarantee of nurse-midwives’ autonomy converges with the premises listed in universal health systems and global institutions such as the World Health Organization, the United Nations and the United Nations Children’s Fund, which treat nursing as the largest workforce in health, with the power to change the care provided to women and their fetuses. Furthermore, in the context described in this study, it will be possible to achieve the freedom and responsibility of nursing professionals through their autonomy, to guarantee dignity in women’s labor and birth, from a management perspective, in the production of important documents.

We emphasized that there is no research available in databases and on the main search engines that reports on nurse-midwives’ autonomy in the implementation of HAA in BC, in the contribution to reaffirming this professional and in achieving freedom of decision within BC. Furthermore, it is important to understand how this professional can play a fundamental role in reducing unfavorable results for women in the labor and birth process, as well as for their conceptus, family and communities, to achieve well-being and health for all, in accordance with the recommendations of the third SDG^(8)^.

## OBJECTIVES

To report nurse-midwives’ autonomy in the implementation of HAI in BCs.

## METHODS

### Ethical aspects

This study was approved by the *Universidade Estadual do Ceará* Research Ethics Committee. The deponent followed the description with total confidentiality and discretion, with no possibility of identification of the deponent or the study sector. The ethical guidelines for research were followed, related to beneficence, non-maleficence and justice, to ensure good practices in research through Resolutions 466/2012 and 510/2016^(9,10)^.

### Study design

This is qualitative research, of the holistic single case study type, carried out in BC located in the northern region of the state of Ceará. The case study provides an approach to numerous facets of social science research, through questions of how and why contemporary phenomena occur within a real-life context, in which their limits may not be formally defined, using strategies such as direct observation and a systematic series of interviews^(11)^. The COnsolidated criteria for REporting Qualitative research (COREQ) was used as a methodological guide for constructing the research^(12)^.

### Study site

The site is a type II intrahospital BC, with five pre-delivery, delivery and post-delivery rooms, linked to the Stork Network since December 2018, which carried out 6,313 services in 2024, being a reference for 11 rural municipalities in a philanthropic institution with majority service to the Brazilian Health System^(13)^. BCs are spaces divided into intrahospital or perihospital, specialized in providing assistance and care for labor, delivery, birth and postpartum of pregnant women and their families. Moreover, these instruments are recognized for their respect, ethics, scientific evidence and female leading role in decision-making regarding health actions^(4)^.

### Data preparation and collection

The case study was constructed based on participant observation carried out by the authors, who are part of the research team for this project, with experience in health management and research development, who worked on the implementation, preparation of human resources for work, and development of activities related to childbirth and birth care for nurse-midwives at the BC. Individual interviews were also conducted with five nurses working at the health facility, who implemented HAA in the service through convenience sampling. The inclusion criteria were minimum six-month experience at the selected BC and specialization in obstetric and/or neonatal nursing. The exclusion criteria were being unemployed at the BC for at least six months and being absent from the location due to vacation or leave. Three agreed to participate in the study, which was conducted at a specific location in the institution, according to the time and date agreed between the researchers and interviewees. Two refused, citing institutional and personal reasons.

### Analysis of cases and between cases

The study refers to events that occurred between the BC implementation in 2018 and the year 2023. A script with four guidelines was followed: how the BC was organized in the hospital; the difficulties at this time; the potential of implementation of HAA by nurse-midwives; and the description of benefits that occurred for maternal and child health in the region. Initially, the reasons for conducting the research were explained to participants, and contacts for interviews occurred through a messaging application at different times during 2020 and 2023. The interviews lasted approximately 20-38 minutes. There were no repeated interviews. For information saturation, the concept of “Information Power” developed by Malterud *et al*. was used, in which the amount of elements contained in the sample information demonstrated its relevance, depending on five points: study objective; sample specificity objective; theory used objective; dialogue quality objective; and analysis strategy employed objective^(14)^.

### Report elaboration

Thematic analysis followed according to Nowell *et al*.^(15)^. The reports were audio-recorded and transcribed in Microsoft Word^®^ for later analysis, following the stages below: I) Familiarization with the information, through reading, triangulation of the different techniques for collecting reports, reflections on all thoughts about the information and identification of potential codes and topics; II) Generation of initial codes, with debates in pairs and use of a coding structure; III) Search for topics through detailed notes on the development and hierarchies of concepts and topics; IV) Review of topics, with topics and subtopics examined by all researchers; V) Definition of topics, through debate in pairs and with all researchers, consensus on the topics and documentation of the findings; VI) Generation of report of topics, through peer assessment of researchers, description of the coding and analysis process, descriptions of context and theoretical basis, and methodological and analytical choices throughout the study. The information was returned through articles to participants, with positive results from participants.

From the organization, the following thematic category was constructed: “Implementation of Hospital Admission Authorization for nurse-midwives’ autonomy in a Birth Center”.

## RESULTS

**Figure 1:**
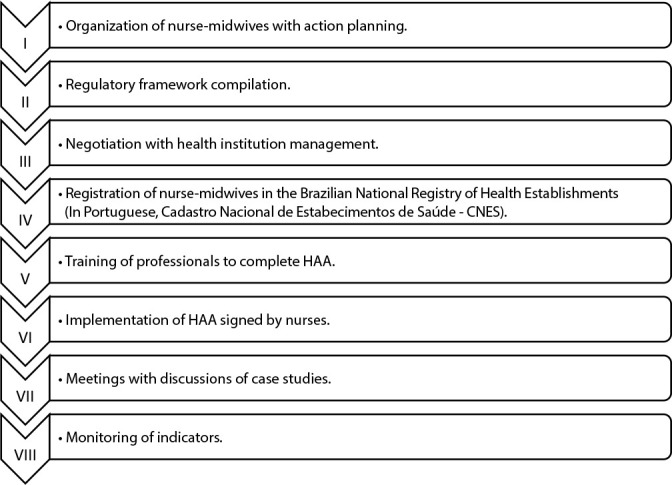
Schedule of activities developed for the implementation of Hospitalization Admission Authorization by nurse-midwives, Fortaleza, Ceará, Brazil, 2024

Figure 1 presents a flowchart of the activities carried out in the implementation of HAA completed and signed by nurse-midwives within the scope of BC.

### Implementation of Hospital Admission Authorization for Nurse-Midwives’ Autonomy in Birth Centers

The hospital highlighted in the study has always been a reference for maternal and child health in the northern region of Ceará; however, it had a high percentage of cesarean sections, which contributed to the city implementing the BC. This incentive is related to public policies to encourage good practices in the obstetric area carried out by the Ministry of Health, based on the Stork Network Strategy, with financial incentives for the implementation of this equipment^(3,4)^.

This scenario is essential for understanding that health policies and government incentives play a key role in the development of care models and in determining the actions of healthcare professionals related to governance, social justice, ethics in care and freedom. In the face of current and long-standing adversities faced in the world, especially in the Global South, such as climate change, hunger, increased mortality from preventable causes and epidemics, Brazil has demonstrated, in the current political situation, significant advances in achieving rights, combating hunger, inclusive policies and participation of Brazilian society in health governance^(16)^.

In the case described, these resources contributed to the construction of a new wing in the hospital, following the models and guidelines recommended by the Ministry of Health for BC in Brazil. However, it was realized that this opportunity could also contribute to changing practices, especially related to nurse-midwives’ autonomy, in accordance with the requirements of the regulations and ordinances that regulate BCs and the nursing profession. Research highlights that there are elements that influence nurses’ autonomous practice, such as structural, cultural and organizational factors, which have an impact on the practice of nurses’ autonomy^(5,17)^.

In order to establish human resources for labor and delivery care, a selection process was conducted to hire nurse-midwives as professionals exclusively dedicated to providing care for labor and delivery without dystocia at the BC where the report was conducted. In addition to this, the BC coordinator discussed with the hospital management the need and importance of nurse-midwives taking charge of the labor process, management, and leadership within the hospital. The autonomy described was related not only to the line of care for labor, delivery, and birth, but also to the management of the health team’s work, management of materials and supplies, and management of HAA dispatch in accordance with required criteria.

All activities were supported by a consultant from the Ministry of Health, who helped develop planned actions that culminated in the implementation of HAA completed and signed by the BC nurse-midwives. Regarding this, a participating nurse said that: “*The incentive that the Ministry provides for the participation of nurse-midwives in the obstetric setting is important for implementing a care model that produces the best outcomes worldwide*”.

From the perspective of a path forward in the face of major global changes, the new conjectures arising from the SDGs imply new actions for nursing aimed at changes in its practices. Increasingly, the responsibility, leadership, autonomy and competency of nursing denote its prominence as an active leading actor in global health, related not only to the large number of nurses and midwives in the health workforce, but also to its relevance and recognition^(18)^.

It is important to highlight that initiatives to implement HAA by nurses within the BC are recent; however, especially in Ceará, there were incentives for this purpose related to the publication of a technical note by the state government that informed and guided municipalities about this possibility according to legislation. One participant reported: “*It was all new to us. We had no clear parameters to follow, no manual that informed us what we should do. We needed to organize ourselves, study, and seek political support internally in the hospital so that we could achieve our goal*”.

In this context, the service management understood the prerogative and importance of this autonomy and freedom of action of nurse-midwives in the development of activities in the healthcare service. To advance this proposal, the institution management and the BC coordinator included qualified nurse-midwives who could sign HAA and that these documents could feed the Hospital Information System and other health information systems.

The next stage in the process of making nurse-midwives’ autonomy effective at the BC was training. It was perceived that there was a need to train nurse-midwives in filling out HAA, since this is not yet a common practice in Brazil. The consultant from the Ministry of Health, a nurse-midwife with experience in filling out HAA, helped in the discussion with professionals so that they could understand the importance of the document, how it was filled out and what it was for. These discussions took place in separate meetings throughout 2018, with case discussions, until HAA could be completed by all nurse-midwives working at the BC.

To perform HAA, nurses should have access to the admission form and fill in spaces with the following data: patient name; brief signs and symptoms of patients’ admission to the unit, such as main complaint, gestational age, date of last menstruation, cervix assessment, fetus presentation, amniotic sac integrity and fetal heartbeat; justification for admission, such as assistance with vaginal delivery; results of tests performed, such as anamnesis and physical examination; initial diagnosis, such as vaginal delivery; nurse-midwife name, signature with stamp and date of the request for admission.

This process ensured that the hospital hosting the BC was able to achieve significant numbers in the completion of HAA by nurse-midwives, in accordance with regulations of the nursing profession and the public health policies directed at maternal and child health in Brazil. Ordinance 11, which guides the implementation of BCs, determines that actions be oriented towards humanized care, governed by regulations, such as a minimum nursing team, with other professionals working in the event of complications, and based on evidence-based practices that have contributed to advances in maternal and child health in Brazil^(2)^.

After implementation, nurses established a working group to discuss cases and strengthen technical activities, especially with the aim of creating a protected and important place to present questions, expand internal partnerships and plan activities by the group. This also enabled monitoring of indicators and continuous improvement of practices based on what was being established in the service’s daily routine.

Encouraging freedom of action and autonomy in nursing practices should not only be a prerogative, but should be based on achieving health and well-being for all, in accordance with the United Nations SDGs. However, advancing actions requires knowledge, technical competency and self-sufficiency to build autonomy, which can only be achieved through specialized training, building professional interactions, and institutional and political incentives^(3,5)^.

The acceptance of management and other healthcare professionals contributed to nurse-midwives gaining prominence in the health unit, based on care production, and BC assistance and management. In this process, women from the health region were referred to the unit and received by nurse-midwives, becoming something common, linked to the routine in the management and conduct of low-risk births. Furthermore, it allowed obstetric nursing in the region to reach a differentiated level with greater visibility, contributing to reducing the maternal mortality rate in relation to other health regions of the state from 2011 to 2021 (0-83.2 versus 119.7-136.9-242) and in the improvement of quality indices in the birth process of women. However, it is important to highlight that, in the Brazilian context, especially in the state of Ceará, it is still a challenge to achieve the target of 30 deaths per 100,000 births of the 2030 Agenda. In 2022, this mortality rate was 74.4 in the state of Ceará, still far from the SDG targets^(19)^.

However, it was assumed in the present study that, in order to change the practices of nurse-midwives and other professionals within BCs, pedagogical changes are necessary in the curriculum, in the critical approach to education and in health training based on interprofessional education and collaborative practices to change professional scenarios in the country and in the world^(17)^. It is worth noting that incentive policies, such as the Stork Network Strategy and Alyne Network, contribute to the valorization of childbirth by nurses with minimal interventions and compromises of maternal rights, dignity and their leading role in birth^(2-4)^. It is recommended that the completion of HAA be included in the content of specializations and residencies in obstetric nursing, with the aim of expanding nurses’ autonomy in the field of work and strengthening the ethical exercise of becoming responsible in the documents for the practices they perform, especially in care for low-risk births.

### Study limitations

The main limitation involved the scarcity of literature on the implementation of HAA by nurse-midwives as well as the need for long-term studies analyzing the repercussions of this activity in Brazilian BCs.

### Contributions to health, nursing or public policy

This manuscript addresses a successful experience in implementing HAA completed by nurse-midwives in BCs, which contributed to the expansion of class organization, autonomy, leadership, decision-making and advances in health management practices of nurse-midwives. These elements represent an improvement in care during labor, delivery and birth, with consequent qualification of care in Brazilian maternal and child health.

## FINAL CONSIDERATIONS

The BC of this study managed to advance women’s and reproductive rights, affirming women’s leading role in decision-making, through institutional incentives and discussion groups, promoted with nurse-midwives as managers of care during childbirth and birth.

The autonomy represented by the completion of HAA by BC nurse-midwives represents a major advance for ethical and healthcare actions in nursing, contributing to effective results in maternal and child health for low-risk pregnant women.

This type of experience reinforces the need for greater autonomy and decision-making power for nurse-midwives, who contribute to improving the health and well-being of individuals, families and global communities.
