# Supplementary material for: Implementation by nurses of Hospital Admission Authorization in Birth Centers
Source: Rev Bras Enferm. 2025 Sep 1;78(Suppl 1):e20240404. doi: 10.1590/0034-7167-2024-0404 (PMC12407879; doi:10.1590/0034-7167-2024-0404)
Supplement: Translation

# Translation: Implementação por enfermeiros da Autorização de Internação Hospitalar em Centros de Parto Normal

**DOI:** 10.1590/0034-7167-2024-0404pt

**Authors:** Antonio Rodrigues Ferreira, Raimundo Neto de Abreu e Brito, Adriano da Costa Belarmino, Fernanda Maria Carvalho Fontenele, Saiwori de Jesus Silva Bezerra dos Anjos, Luiza Jane Eyre de Souza Vieira

**Affiliations:** I Universidade Estadual do Ceará. Fortaleza, Ceará, Brasil; II Hospital e Maternidade Dr José Maria Fernandes Leitão. Novo Oriente, Ceará, Brasil; III Universidade de Fortaleza. Fortaleza, Ceará, Brasil

**Keywords:** Enfermeiros Obstetras, Centros de Assistência à, Gravidez e ao Parto, Papel Profissional, Autonomia Profissional, Tomada de Decisão

## Abstract

**Objetivos:**

relatar a autonomia de enfermeiros obstetras na implementação da Autorização de Internação Hospitalar em Centros de Parto Normal.

**Métodos:**

trata-se de estudo de caso em Centros de Parto Normal intrahospitalares no Ceará, Brasil, de eventos ocorridos entre 2018 e 2023. A organização da descrição seguiu as etapas da análise temática conforme Nowell.

**Resultados:**

a autonomia e a liberdade de atuação do enfermeiro obstetra nas práticas de cuidado e gestão no trabalho de parto, parto e nascimento propiciam o protagonismo da categoria profissional no cenário obstétrico, ressaltando sua necessidade para garantir direitos femininos e minorar complicações materno-fetais.

**Considerações Finais:**

a emissão de Autorização de Internação Hospitalar pelos enfermeiros obstetras em Centros de Parto Normal representa avanço significativo para as ações éticas e de cuidado em saúde da equipe de enfermagem, contribuindo para resultados efetivos em saúde materno-infantil e alcance de progressos na prática avançada na profissão da enfermagem obstetra.

## INTRODUÇÃO

Garantir a mortalidade materna menor que 70 por 100 mil nascidos vivos segue como um dos maiores desafios dos Objetivos de Desenvolvimento Sustentável (ODS) no mundo, principalmente após a pandemia de COVID-19. Em 2020, 800 mulheres morreram por dia no mundo, associado principalmente às causas evitáveis e intervenções desnecessárias ao parto e nascimento, como a via de nascimento cirúrgica sem indicação. Cerca 95% desses óbitos foram em países de média e baixa renda, como o Brasil^(1)^.

Nessa perspectiva, os modelos de atenção à saúde, como a Estratégia Rede Cegonha e a Rede Alyne, desempenham papel primordial na garantia de direitos, justiça social, equidade no acesso, no cuidado e na assistência em saúde materno-infantil no país^(2,3)^. No componente Parto e Nascimento da Rede Cegonha e Rede Alyne, os Centros de Parto Normal (CPNs) vêm desenvolvendo modelos de atenção ao parto que primam por práticas baseadas em evidências científicas, ambiência e estruturas direcionadas a garantir familiaridade e conforto, equipe profissional capacitada e ações que garantem o direito da mulher ao parto e nascimento com qualidade, assim como seu protagonismo e tomada de decisão nos processos de cuidado^(2)^.

Nesse contexto, o enfermeiro obstetra se destaca como promotor de assistência, cuidado e gestão do trabalho nos CPNs, incentivado por políticas públicas de saúde e pela sociedade. No entanto, para que isso ocorra, ainda se enfrentam empecilhos e obstáculos relacionados às disputas profissionais e de poder dentro do modelo biomédico e hospitalocêntrico de atenção ao parto e nascimento vigente no país^(4,5)^.

Uma das competências essenciais para atuação dentro desses espaços e superação dos obstáculos refere-se à autonomia para as ações em saúde. Entre elas, está a implementação da Autorização de Internação Hospitalar (AIH) de mulheres em trabalho de parto e parto, o que é permitido legalmente ao enfermeiro obstetra. A AIH é um documento que contém os dados acerca do usuário do sistema de saúde brasileiro, que alimenta os sistemas de informações hospitalar e ambulatorial para produção de indicadores^(4,6)^.

Relativo a isso, o conceito adotado de autonomia se refere a habilidades e técnicas, como conhecimento, ausência de dependência de outros, poder de decisão, competência, autogovernança, liberdade, capacidade de afeto, julgamento, autocontrole e responsabilidade^(7)^. A garantia de autonomia de enfermeiros obstetras converge com as premissas elencadas nos sistemas de saúde universais e de instituições globais como a Organização Mundial da Saúde, a Organização das Nações Unidas e o Fundo das Nações Unidas para Infância, que tratam a enfermagem como a maior força de trabalho na saúde, com poder de modificar a atenção à mulher e seu concepto. Ademais, no contexto descrito no presente estudo, poder-se-á alcançar a liberdade e responsabilidade dos profissionais de enfermagem através de sua autonomia, para garantir dignidade no parto e nascimento de mulheres, a partir de uma perspectiva da gestão, na produção de documentos importantes.

Ressaltamos que não há pesquisas disponíveis em bases de dados e nos principais sites de busca que relatem a autonomia do enfermeiro obstetra na implementação da AIH no CPN, na contribuição para reafirmar esse profissional e na conquista da liberdade de decisão dentro do CPN. Ademais, é relevante entender como este pode desempenhar papel fundamental na diminuição de resultados desfavoráveis para a mulher no processo de parto e nascimento, assim como para seu concepto, família e comunidades, para o alcance de bem-estar e saúde para todos, conforme as recomendações do terceiro ODS^(8)^.

## OBJETIVOS

Relatar a autonomia de enfermeiros obstetras na implementação da AIH em CPNs.

## MÉTODOS

### Aspectos éticos

O presente estudo obteve aprovação do Comitê de Ética em Pesquisa da Universidade Estadual do Ceará. O depoente seguiu a descrição com total sigilo e discrição, com impossibilidade de identificação do mesmo e do setor de estudo. As diretrizes éticas de pesquisa foram seguidas, relacionadas à beneficência, não maleficência e justiça, para garantir as boas práticas em pesquisas por meio das Resoluções n° 466/2012 e n° 510/2016^(9,10)^.

### Desenho do estudo

Trata-se de pesquisa de cunho qualitativo, do tipo estudo de caso único holístico, realizado em CPN localizado na região Norte do estado do Ceará. O estudo de caso proporciona a abordagem de inúmeras facetas da pesquisa em ciências sociais, através de questionamentos de como e porque fenômenos contemporâneos acontecem dentro de um contexto da vida real, em que seus limites podem não estar formalmente definidos, utilizando estratégias como observação direta e série sistemática de entrevistas^(11)^. O *COnsolidated criteria for REporting Qualitative research* (COREQ) foi utilizado como roteiro metodológico para construção da pesquisa^(12)^.

### Local do estudo

O local é um CPN intrahospitalar do tipo II, com cinco salas de pré-parto, parto e pós-parto, vinculado à Rede Cegonha desde dezembro de 2018, que realizou 6.313 atendimentos em 2024, sendo referência para 11 municípios sertanejos em instituição filantrópica com atendimento majoritário ao Sistema Único de Saúde^(13)^. Os CPNs são espaços divididos em intrahospitalar ou perihospitalar, especializados na assistência e cuidado ao trabalho de parto, parto, nascimento e puerpério de mulheres gestantes e suas famílias. Além disso, esses instrumentos são reconhecidos pelo respeito, ética, evidências científicas e protagonismo feminino na tomada de decisão das ações de saúde^(4)^.

### Preparação e coleta dos dados

O estudo de caso foi construído a partir da observação participante realizada pelos autores, que compõem a equipe de pesquisa deste construto, com experiência em gestão em saúde e desenvolvimento de pesquisas que atuaram na implantação, preparação dos recursos humanos para o trabalho e desenvolvimento das atividades referentes ao cuidado ao parto e nascimento dos enfermeiros obstetras no CPN. Também foram realizadas entrevistas individuais com cinco enfermeiros atuantes no equipamento de saúde, que implementaram a AIH no serviço através de amostra por conveniência. Os critérios de inclusão foram a atuação mínima de seis meses no CPN selecionado e possuir especialização em enfermagem obstétrica e/ou neonatal. Como critérios de exclusão, foram empregadas a desvinculação com o CPN há pelo menos seis meses e a ausência no local devido a férias ou licença. Três concordaram em participar do estudo, sendo realizado em local específico da instituição, conforme horário e data pactuados entre os pesquisadores e os entrevistados. Dois recusaram, referindo razões institucionais e pessoais.

### Análise dos casos e entre os casos

O estudo é referente aos eventos ocorridos entre a implantação do CPN, em 2018, e o ano de 2023. Seguiu-se um roteiro com quatro orientações: como ocorreu a organização do CPN no hospital; as dificuldades neste momento; as potencialidades da implantação da AIH pelos enfermeiros obstetras; e a descrição dos benefícios que ocorreram para saúde materno-infantil na região. Inicialmente, as razões para realização da pesquisa foram explicadas aos participantes, e os contatos para as entrevistas ocorreram por meio de aplicativo de mensagens em momentos diferentes durante o ano de 2020 e 2023. As entrevistas tiveram duração de cerca de 20-38 minutos. Não ocorreram entrevistas repetidas. Para a saturação das informações, utilizou-se o conceito de “Poder da Informação” desenvolvido por Malterud *et al*., em que a quantidade de elementos contidos nas informações da amostra demonstrou sua relevância, dependendo de cinco pontos: o objetivo do estudo; o objetivo da especificidade da amostra; o objetivo da teoria utilizada; o objetivo da qualidade dos diálogos; e o objetivo da estratégia de análise empregada^(14)^.

### Elaboração dos relatórios

Seguiu-se a análise temática conforme Nowell *et al*.^(15)^. Os relatos foram audiogravados e transcritos em *Microsoft Word^®^* para posterior análise seguindo as etapas a seguir: I) Familiarização com as informações, através de leitura, triangulação das diferentes técnicas de coleta dos relatos, reflexões sobre todos os pensamentos acerca das informações e identificação de potenciais códigos e temas; II) Geração dos códigos iniciais, com debates em pares e utilização de uma estrutura de codificação; III) Procura por temas através da anotação detalhada de desenvolvimento e hierarquias de conceitos e temas; IV) Revisão dos temas, com temas e subtemas examinados por todos os pesquisadores; V) Definição dos temas, por meio do debate em pares e com todos os pesquisadores, consenso em relação aos temas e documentação dos achados; VI) Geração do relatório dos temas, através de avaliação por pares dos pesquisadores, descrição do processo de codificação e análise, descrições do contexto e da fundamentação teórica, das escolhas metodológicas e analíticas durante todo o estudo. As informações foram devolvidas através de artigos para os participantes, com resultados positivos dos participantes.

A partir da organização, construiu-se a seguinte categoria temática “A implementação da Autorização de Internação Hospitalar para autonomia do enfermeiro obstetra em Centro de Parto Normal”.

## RESULTADOS

Apresenta-se na Figura 1 um fluxograma das atividades realizadas na implementação da AIH preenchida e assinada por enfermeiros obstetras no âmbito de CPN.

**Figura 1:** Cronograma das atividades desenvolvidas para implementação da Autorização de Internação Hospitalar por enfermeiros obstetras, Fortaleza, Ceará, Brasil, 2024

### A implementação da Autorização de Internação Hospitalar para autonomia do enfermeiro obstetra em Centros de Parto Normal

O hospital em destaque no estudo sempre foi referência para saúde materno-infantil na região Norte do Ceará, no entanto apresentava um percentual elevado de cesáreas, o que contribuiu para que o município implantasse o CPN. Esse incentivo está relacionado às políticas públicas de indução de boas práticas na área obstétrica realizadas pelo Ministério da Saúde, a partir da Estratégia Rede Cegonha, com incentivos financeiros para implementação deste equipamento^(3,4)^.

Esse cenário é essencial para o entendimento de que as políticas de saúde e incentivos governamentais desempenham fator primordial para o desenvolvimento de modelos de atenção e para determinar as ações dos profissionais de saúde relacionadas à governança, justiça social, ética no cuidado e liberdade. Diante das atuais e antigas adversidades enfrentadas no mundo, principalmente pelo Sul Global, como mudanças climáticas, fome, aumento de mortalidade por causas evitáveis e epidemias, o Brasil vem demonstrando, na atual conjuntura política, avanços significativos no alcance de direitos, combate à fome, políticas inclusivas e participação da sociedade brasileira na governança em saúde^(16)^.

No caso descrito, esses recursos contribuíram para construção de uma nova ala no hospital, seguindo os modelos e diretrizes preconizadas pelo Ministério da Saúde para CPN no Brasil. No entanto, percebeu-se que essa oportunidade poderia contribuir também para mudança de práticas, especialmente relacionadas à autonomia do enfermeiro obstetra, conforme as exigências das normativas e portarias que regulamentam os CPNs e a profissão de enfermagem. Pesquisas ressaltam que há elementos que influenciam a prática autônoma do enfermeiro, como fatores estruturais, culturais e organizacionais, repercutindo na prática de autonomia dos enfermeiros^(5,17)^.

A fim de constituir os recursos humanos direcionados ao cuidado ao parto e nascimento, ocorreu uma seleção para contratação de enfermeiros obstetras como profissionais exclusivos no cuidado ao parto sem distocia no CPN sede do relato. Além disso, ocorreu o diálogo do coordenador do CPN com a direção do hospital acerca da necessidade e importância de os enfermeiros obstetras assumirem o processo parturitivo, a gestão e a liderança dentro do local. A autonomia descrita era relacionada não somente à linha de cuidado ao trabalho de parto, parto e nascimento, mas também à gestão do trabalho da equipe de saúde, gestão de materiais e insumos, e gestão da expedição da AIH diante dos critérios exigidos.

Todas as atividades contaram com apoio de consultor do Ministério da Saúde, que auxiliou no desenvolvimento das ações planejadas que culminaram com a implementação da AIH preenchida e assinada pelos enfermeiros obstetras do CPN. Acerca disso, um enfermeiro participante diz que: “*A indução que o Ministério propicia para a participação do enfermeiro obstetra no cenário obstétrico é importante para implementarmos um modelo de atenção que mundialmente produz os melhores desfechos*”.

Na perspectiva de um caminho para seguir diante das grandes alterações globais, as novas conjecturas decorrentes dos ODS implicam novas ações da enfermagem direcionadas a mudanças nas suas práticas. Cada vez mais, a responsabilidade, liderança, autonomia e competência da enfermagem denotam seu destaque como ativo protagonista na saúde global, relacionado não somente ao grande número de enfermeiros e parteiros na força de trabalho em saúde, mas aliado à sua relevância e reconhecimento^(18)^.

É importante destacar que as iniciativas de implementação de AIH por enfermeiros no âmbito do CPN são recentes, no entanto, especialmente no Ceará, ocorreram incentivos para esse propósito relacionados à publicação de uma nota técnica do governo estadual que informava e orientava os municípios acerca dessa possibilidade conforme a legislação. Um participante relatou: “*Foi tudo novo para nós. Não tínhamos parâmetros claros para seguir, nenhum manual que informasse o que deveríamos fazer. Precisamos nos organizar, estudar, buscar apoio político internamente no hospital para que alcançássemos nosso objetivo*”.

Nesta conjuntura, a direção do serviço entendeu a prerrogativa e a importância dessa autonomia e liberdade de atuação dos enfermeiros obstetras no desenvolvimento das atividades no serviço de saúde. Para avançar nessa proposta, a direção da instituição e o coordenador do CPN incluíram os enfermeiros obstetras habilitados que pudessem assinar a AIH e que estes documentos pudessem alimentar o Sistema de Informação Hospitalar e outros sistemas de informação em saúde.

A próxima etapa no processo de efetivação da autonomia dos enfermeiros obstetras no CPN foi a capacitação. Percebeu-se a necessidade de formação de enfermeiros obstetras no preenchimento da AIH, visto que ainda não é prática comum no Brasil. O consultor do Ministério da Saúde, enfermeiro obstetra com experiência no preenchimento da AIH, auxiliou na discussão com os profissionais, para que pudessem perceber a importância do documento, como ocorria seu preenchimento e para que servia. Essas discussões ocorreram em reuniões distintas no decorrer do ano de 2018, com discussão de casos, até que pudesse ser implementado o preenchimento de AIH por todos os enfermeiros obstetras atuantes no CPN.

Para realização da AIH, o enfermeiro deveria ter acesso ao formulário de internação e preencher os espaços com os seguintes dados: nome da paciente; breve sinais e sintomas da admissão da paciente na unidade, como queixa principal, idade gestacional, data da última menstruação, avaliação do colo uterino, apresentação do feto, integridade da bolsa amniótica e batimentos cardiofetais; justificativa de internação, como assistência ao parto normal; resultados de exames realizados, como anamnese e exame físico; diagnóstico inicial, como parto normal; nome do enfermeiro obstetra, assinatura com carimbo e data da solicitação do internamento.

Esse processo garantiu que o hospital sede do CPN conseguisse alcançar números consideráveis no preenchimento da AIH pelos enfermeiros obstetras, conforme as regulamentações da profissão de enfermagem e as próprias políticas de saúde pública direcionadas à saúde materno-infantil no Brasil. A Portaria nº 11, que orienta a implantação dos CPNs, determina que as ações sejam orientadas à atenção humanizada, regida por normativas, como equipe mínima de enfermagem, com outros profissionais atuantes, diante de intercorrências, e alicerçada em práticas baseadas em evidências que têm contribuído para avanços na saúde materno-infantil no Brasil^(2)^.

Após a implementação, os enfermeiros instituíam um grupo de trabalho para discussão de casos e fortalecimento técnico das atividades, especialmente com o intuito de construir um local protegido e importante para a apresentação de dúvidas, ampliação das parcerias internas e planejamento das atividades pelo coletivo. Isso também propiciou o monitoramento dos indicadores e a qualificação contínua das práticas a partir do que estava sendo instituído no cotidiano do serviço.

O incentivo à liberdade de atuação e autonomia nas práticas da enfermagem não somente deve ser uma prerrogativa, mas deve estar alicerçado no alcance de saúde e bem-estar para todos, conforme os ODS das Nações Unidas. Entretanto, avançar nas ações exige conhecimento, competência técnica e autossuficiência para construção de autonomia que somente pode ser alcançado por meio de formação especializada, construção de interações profissionais, além de incentivos institucionais e políticos^(3,5)^.

A aceitação da direção e dos demais profissionais de saúde contribuiu para que os enfermeiros obstetras ganhassem destaque na unidade de saúde, a partir da produção de cuidado, na assistência e na gestão do CPN. Neste processo, mulheres oriundas da região de saúde eram encaminhadas à unidade e recebidas pelo enfermeiro obstetra, tornando-se algo comum, concatenado à rotina no manejo e condução de partos de risco habitual. Ademais, permitiu que a enfermagem obstétrica na região alcançasse um patamar diferenciado com maior visibilidade, contribuindo na diminuição da taxa de mortalidade materna com relação às outras regiões de saúde do estado de 2011-2021 (0-83,2 contra119,7-136,9-242) e na melhoria dos índices de qualidade no processo parturitivo de mulheres. No entanto, é importante destacar que, no contexto brasileiro, especialmente no estado do Ceará, ainda é um desafio conseguir alcançar a meta de 30 mortes por 100 mil nascimentos da Agenda 2030. Em 2022, essa razão de mortalidade era de 74,4 no estado do Ceará, ainda distante das metas dos ODS^(19)^.

No entanto, assumiu-se no presente estudo que, para mudança de práticas dos enfermeiros obstetras e demais profissionais dentro dos CPNs, são necessárias modificações pedagógicas no currículo, na abordagem crítica da educação e na formação em saúde alicerçadas em educação interprofissional e práticas colaborativas para mudança de cenários profissionais no país e no mundo^(17)^. Salienta-se que políticas de incentivo, como a Estratégia Rede Cegonha e Rede Alyne, contribuem para valorização do parto pelo enfermeiro com o mínimo de intervenções e comprometimentos do direito, dignidade materna e de seu protagonismo no nascimento^(2-4)^. Recomenda-se que o preenchimento da AIH seja incluído no conteúdo das especializações e residências em enfermagem obstétrica, com o intuito de ampliar a autonomia do enfermeiro no campo de trabalho e fortalecer o exercício ético de se tornar responsável nos documentos pelas práticas que executa, especialmente na atenção ao parto de risco habitual.

### Limitações do estudo

A principal limitação envolveu a escassez de literatura acerca da implementação de AIH por enfermeiros obstetras, bem como a necessidade de estudos de longo prazo com análise das repercussões desta atividade nos CPN brasileiros.

### Contribuições para as áreas da saúde, enfermagem ou políticas públicas

O presente manuscrito aborda uma experiência exitosa na implementação da AIH preenchida por enfermeiros obstetras em CPN, o que contribuiu para ampliação da organização de classe, autonomia, liderança, tomada de decisão e avanços nas práticas na gestão em saúde do enfermeiro obstetra. Esses elementos representam melhoria do cuidado ao trabalho de parto, parto e nascimento, com consequente qualificação da atenção na saúde materno-infantil brasileira.

## CONSIDERAÇÕES FINAIS

O CPN do estudo conseguiu avançar nos direitos femininos e reprodutivos, afirmando o protagonismo das mulheres na tomada de decisão, através de incentivos institucionais e grupos de discussão, fomentados com os enfermeiros obstetras como gestores do cuidado ao parto e nascimento.

A autonomia representada pelo preenchimento da AIH pelos enfermeiros obstetras de CPN significa grande avanço para as ações éticas e de cuidado em saúde da enfermagem, contribuindo para resultados efetivos em saúde materno-infantil de gestantes de risco habitual.

Este tipo de experiência reforça a necessidade de maior autonomia e poder de decisão dos enfermeiros obstetras, que contribuem para melhoria da saúde e bem-estar dos indivíduos, famílias e comunidades globais.
